# Nucleic acid delivery to mesenchymal stem cells: a review of nonviral methods and applications

**DOI:** 10.1186/s13036-019-0140-0

**Published:** 2019-01-18

**Authors:** Andrew Hamann, Albert Nguyen, Angela K. Pannier

**Affiliations:** Department of Biological Systems Engineering, University of Nebraska-Lincoln, 231 L.W. Chase Hall, Lincoln, NE 68583-0726 USA

**Keywords:** Nonviral gene delivery, Transfection, Human mesenchymal stem cells, Cell therapy, Gene therapy, Priming

## Abstract

**Background:**

Mesenchymal stem cells (MSCs) are multipotent stem cells that can be isolated and expanded from many tissues, and are being investigated for use in cell therapies. Though MSC therapies have demonstrated some success, none have been FDA approved for clinical use. MSCs lose stemness *ex vivo,* decreasing therapeutic potential, and face additional barriers *in vivo,* decreasing therapeutic efficacy. Culture optimization and genetic modification of MSCs can overcome these barriers. Viral transduction is efficient, but limited by safety concerns related to mutagenicity of integrating viral vectors and potential immunogenicity of viral antigens. Nonviral delivery methods are safer, though limited by inefficiency and toxicity, and are flexible and scalable, making them attractive for engineering MSC therapies.

**Main text:**

Transfection method and nucleic acid determine efficiency and expression profile in transfection of MSCs. Transfection methods include microinjection, electroporation, and nanocarrier delivery. Microinjection and electroporation are efficient, but are limited by throughput and toxicity. In contrast, a variety of nanocarriers have been demonstrated to transfer nucleic acids into cells, however nanocarrier delivery to MSCs has traditionally been inefficient. To improve efficiency, plasmid sequences can be optimized by choice of promoter, inclusion of DNA targeting sequences, and removal of bacterial elements. Instead of DNA, RNA can be delivered for rapid protein expression or regulation of endogenous gene expression. Beyond choice of nanocarrier and nucleic acid, transfection can be optimized by priming cells with media additives and cell culture surface modifications to modulate barriers of transfection. Media additives known to enhance MSC transfection include glucocorticoids and histone deacetylase inhibitors. Culture surface properties known to modulate MSC transfection include substrate stiffness and specific protein coating. If nonviral gene delivery to MSCs can be sufficiently improved, MSC therapies could be enhanced by transfection for guided differentiation and reprogramming, transplantation survival and directed homing, and secretion of therapeutics. We discuss utilized delivery methods and nucleic acids, and resulting efficiency and outcomes, in transfection of MSCs reported for such applications.

**Conclusion:**

Recent developments in transfection methods, including nanocarrier and nucleic acid technologies, combined with chemical and physical priming of MSCs, may sufficiently improve transfection efficiency, enabling scalable genetic engineering of MSCs, potentially bringing effective MSC therapies to patients.

## Background

### Human mesenchymal stem cells (hMSCs)

Human mesenchymal stem cells (hMSCs) are multipotent adult stem cells that can be easily isolated and expanded from many tissues, including bone marrow (hBMSCs), adipose (hAMSCs), and umbilical cord (hUCMSCs) [1]. hMSCs are tri-lineage multipotent in vitro (osteogenic, adipogenic, and chondrogenic) [2], and home to sites of inflammation in damaged tissues in vivo after transplantation [3], where they can facilitate tissue repair through differentiation for cell repopulation, and promote tissue remodeling and modulation of the immune response through secretion of growth factors, cytokines, and exosomes [4–12]. hMSCs are immune evasive [13], enabling allogenic transplantation for cell therapies that make use of the aforementioned properties. Additionally, MSCs can be efficiently reprogrammed to generate induced pluripotent stem cells (iPSCs) [14, 15]. Therefore, hMSCs are being widely investigated for use in cell therapies for treatment of many diseases.

Over 200 hMSC cell therapy clinical trials have been completed, and another 81 are currently active, according to the US National Library of Medicine clinical trial database. These clinical trials include therapies for treatment of autoimmunity, graft versus host disease, ischemia, injury of central nervous system, and cancer [16]. Though clinical trials have demonstrated some measures of success, no hMSC therapy has been approved by the FDA for clinical use [17]. hMSC therapies have not resulted in widespread success, in part due to challenges associated with maintenance of stemness during expansion ex vivo, resulting in progressive loss of self-renewal, differentiation potential, and immunomodulatory capacity that decrease hMSC therapeutic potential, as well as additional challenges after delivery in vivo, including transplantation survival and target engraftment [18–20]. To overcome the barriers that limit their performance in therapies, and enhance their properties, hMSCs can be modified by optimization of culture conditions and exogenous gene transfer, ex vivo. In expansion, stemness maintenance can be enhanced by inclusion of media factors [21–24], and tuning of substrate properties or culturing in 3-D [25–27], in order to suppress cell senescence. Loss of proliferative capacity, pluripotent gene expression, and differentiation potential in MSCs are, in part, due to senescence linked to low telomerase activity [28, 29]. Through gene delivery, MSCs have been successfully immortalized by induced expression of human telomerase reverse transcriptase (hTERT), which significantly extends MSC expansion before replicative senescence, while retaining expression of pluripotency genes, immunosuppressive properties, and differentiation potential [30, 31]. hMSCs can also be engineered ex vivo to enhance therapeutic function in vivo, through induced expression of pro-survival genes [32, 33], adhesion ligands targeting cell membrane receptors [34–36], lineage-specific genes for directed differentiation [37, 38], or genes that encode for production and secretion of growth factors [39, 40], cytokines [41, 42] and miRNA in exosomes [43, 44]. Thus, many researchers are investigating methods to efficiently transfer genes to MSCs.

### Gene delivery to hMSCs

Gene delivery is the delivery of exogenous DNA or RNA to cells to alter gene expression. The primary challenges of gene delivery are efficiency and safety. Viral gene delivery systems use the naturally efficient mechanisms of viruses to condense nucleic acids and mediate their internalization, trafficking, and expression within target cells [45]. In general, these vehicles can be engineered by addition of exogenous genes and removal of deleterious viral genes to render replication-deficiency and decrease pathogenicity [45]. Viral gene delivery, while highly efficient, is limited by safety issues, including insertional mutagenicity. Specifically, a clinical trial in which hematopoietic stem cells (HSCs) were genetically modified with retrovirus prior to transplantation resulted in four patients developing leukemia due to insertional mutagenic transformation [16]. Though MSCs have thus far not been found tumorigenic in clinical trials [46, 47], transduction with viral vectors could increase this risk. Even with viral vectors that do not integrate, safety risks of viral transduction in the manufacture of cell therapies remain due to possible presentation [48] of viral antigens on transduced cells that could potentially activate an immune response in vivo following transplantation [49]. In addition, viral systems are limited by relatively small transgene cargo capacity, and difficulty in production and scale-up [50]. Safety risks and manufacturing challenges motivate the development of methods for efficient nonviral gene delivery to hMSCs. Nonviral gene delivery methods are safer than viral methods, and are more scalable and flexible, but are less efficient and can be toxic, especially in hMSCs. This review will discuss current methods and materials for nonviral gene delivery to MSCs, approaches that improve transfection efficiency with MSC priming by media additives and culture surface design, and potential clinical applications that utilize nonviral gene delivery to MSCs.

## Methods of nonviral nucleic acid delivery into MSCs ex vivo

In nonviral gene transfer, delivery method and choice of nucleic acid will determine transfection outcome. In this section, we review microinjection, electroporation, and nanocarriers as methods of nucleic acid delivery, highlight DNA sequence considerations, compare delivery efficiencies of mRNA versus DNA cargoes, and discuss utility of oligonucleotide delivery, in the context of MSCs.

### MSC transfection via membrane disruption by microinjection and electroporation

Effective strategies for nonviral transfection of MSCs ex vivo typically employ disruption of cell membranes to transfer nucleic acids into cells (e.g. microinjection, electroporation) or packaging of nucleic acids with nanocarrier materials that facilitate cellular internalization through endocytosis [51]. For both membrane disruption- and nanocarrier-mediated delivery, intracellular barriers remain following nucleic acid delivery to the cytoplasm, including lysosomal and nuclease degradation, and for plasmid DNA (pDNA), cytoplasmic transport to and import through the nuclear membrane into the nucleus [51]. Nuclear localization of plasmid is eased in proliferative cell types due to dissolution of the nuclear membrane during mitosis; conversely, nuclear localization of plasmids is challenging in primary cell types such as MSCs, which proliferate slowly and are hard-to-transfect [52]. Thus, microinjection of plasmids into MSCs is efficient when delivered directly into the nucleus, demonstrated in single or few MSCs with nanoneedles 200–275 nm in diameter, with 75% viability retained after injection and 65–75% reporter transgene expression efficiency (i.e. percentage of cells expressing transgene) [53, 54]. However, microinjection is impractical for transfecting large numbers of cells.

Electroporation is a higher throughput alternative to microinjection that applies transient electric fields to cell populations, typically in suspension, inducing pores in cell membranes that allow entry of nucleic acids into the cytoplasm [55], though induced charge association between nucleic acids and cell membranes followed by endocytosis has been demonstrated as an alternative mechanism [56]. Electroporation is economical (not necessarily requiring additional reagents beyond suspension buffer) and is widely used for DNA and RNA transfection of MSCs with high efficiency, as described below [57], though as with microinjection, nuclear localization is a primary barrier for plasmid delivery via electroporation [58]. A commercial electroporation system known as Nucleofector (Lonza, Germany) employs cell-type specific electric field pulse parameters and proprietary suspension solution formulations to drive plasmid DNA transfer directly to the nucleus, a method termed nucleofection [59]. Nucleofection of MSCs has been demonstrated to increase transfection efficiency of plasmid delivery relative to conventional electroporation [60], with approximately 68% transfection efficiency in MSCs electroporated in Nucleofector buffer suspension and subsequently re-plated [61], however cell viability after nucleofection was reported as 54%.

While effective at transfection, as reported above, electroporation is limited by cytotoxicity, which is attributed to effects of the pulsed electric fields on biomolecules, including electroconformation change of lipid membranes, proteins, and DNA, and oxidative damage from generated reactive oxygen species [55]. Additional mechanisms of electroporation cytotoxicity include Joule heating, pH change, and metal ions electrolytically shed from aluminum, copper, or stainless-steel electrodes into the electroporation buffer solution [55]. The Neon (Thermo Fisher Scientific, USA) micro-electroporation system uses long, narrow, low volume capillaries, as opposed to traditional electroporation cuvettes, decreasing changes in pH, and allowing for economical use of small gold electrodes that are more electrolytically inert [62]. Using micro-electroporation (notably with an earlier version of the Neon system), plasmid delivery to hUCMSCs achieved approximately 80% transfection efficiency and 80% viability [63]. However, while electroporation methods are efficient, current laboratory-scale electroporation systems are low-throughput. Clinical application of electroporation to MSCs will require significant scale-up that maintains high transfection efficiency and achieves high viability, which remains a challenge. Nanocarriers described below are a potentially more scalable approach for nonviral gene delivery to MSCs.

### MSC transfection via nanocarrier materials

The primary alternative to electroporation for nucleic acid transfer into MSCs ex vivo is transfection with nanocarriers, materials that electrostatically condense or encapsulate nucleic acids into nanoparticles or aggregate complexes that favorably associate with cell membranes through charge interactions or surface receptor binding, and are subsequently internalized via macropinocytosis, clathrin-mediated endocytosis, or caveolae-mediated endocytosis, depending primarily on nanoparticle size and charge [64]. Generally, size and charge of nanoparticles can be tuned by varying the ratio of nucleic acid to nanocarrier formulation. A wide variety of carriers have been demonstrated to facilitate transfection of MSCs, including polymers, lipids, polysaccharides, peptides, and inorganic materials [65].

Cationic lipids are commonly used for transfection of MSCs, for example, Hoare et al. [66] transfected hBMSCs with pDNA encoding for enhanced green fluorescent protein (EGFP) complexed with the commercially available cationic lipid transfection reagent, Lipofectamine (LF) 2000. Transfection efficiency increased from 20 to 40% and viability decreased from 80 to 50%, as the lipid/pDNA (*v*/*w*) ratio increased from 5 to 20, respectively. A newer version of LF, LF-LTX, was used by Kelly et al. [67] and achieved 2–6% transfection efficiency after 48 h in hBMSCs with significantly decreased metabolic activity compared to untransfected hBMSCs, while the latest LF iteration, LF3000, achieves up to 26% transfection efficiency in hBMSCs, according to de Carvalho et al., though viability was not reported [68].

For comparison of several other types of nanocarriers, Gonzalez-Fernandez et al. [69] tested biocompatible mineral nano-hydroxyapatite (nHA), the ubiquitous cationic polymer transfection reagent 25 kDa branched polyethylenimine (bPEI), and repeating arginine–alanine-leucine-alanine (RALA) amphipathic peptide for porcine BMSC transfection with pDNA encoding GFP. All three nanocarriers exhibited transfection efficiency between 15 and 20% and metabolic activity was not significantly decreased after 3 days, with the exception of PEI, which exhibited a 30% decrease in metabolic activity relative to untransfected control.

While nanocarriers, in general, have thus far not achieved transfection efficiency in MSCs that competes with electroporation or viral vectors while maintaining high viability, through flexibility of design and chemical modification, continuous improvements to nanocarriers are being developed. For example, nanocarriers can be functionalized with ligands to facilitate cellular uptake of nanoparticles. To improve nanoparticle uptake by MSCs, Saraf et al. [70] functionalized bPEI with hyaluronic acid, which binds to MSC surface receptor CD44, and this modified bPei was used to deliver pDNA encoding EGFP to hMSCs, resulting in 3-fold higher transfection efficiency than unfunctionalized bPEI transfection. In a similar approach, Santos et al. [71] functionalized a commonly used gene delivery dendrimer, poly(amidoamine) (PAMAM), with proprietary peptides that were previously reported to target MSCs with high affinity. The targeting peptides significantly increased plasmid uptake by hMSCs and increased luciferase transgene expression 10-fold after 48 h, without significantly decreasing cell viability, relative to unmodified PAMAM. To promote transport to nuclei, nuclear localization sequences (NLS), which are found on transcription factors and facilitate their transport into nuclei [72], have been incorporated into nanocarriers. For example, Hoare et al. [66] incorporated NLS peptides into LF2000-pDNA complexes, and demonstrated hBMSC luciferase transgene expression was increased about 10-fold (varying between different NLS), relative to hBMSC transgene expression mediated by lipoplexes without an NLS.

The highest transfection efficiency reported in the literature for transfection of MSCs via nanocarriers has been achieved by biocompatible and degradable poly(β-amino-esters) (PBAE). In Mangraviti et al. [73], a library of PBAEs was screened in high-throughput to determine which polymers could mediate high transfection without decreased viability in hAMSCs. The highest-performing PBAE, PBAE_536e_, had a molecular weight of 8.5 kDa, and when mixed with DNA at a 40:1 ratio by mass, achieved 75% transfection efficiency and 71% viability. These PBAE nanocarriers achieve transfection efficiency similar to, and viability higher than, optimized electroporation methods, and are therefore promising candidates for scaling nonviral gene delivery to MSCs for clinical applications.

While these studies demonstrate that nanocarriers have the potential to be achieve high transfection efficiency, it should be noted that vast differences in transfection outcomes are reported, presumably due to variability in species, tissue source, passage, and donor of the MSCs. MSCs are universally characterized by expression of specific surface markers (CD73+, CD90+, CD105+), but expression of pluripotency transcription factors and surface markers varies between tissue sources, and donors [74], resulting in differences in proliferative capacity [75], differentiation potential [75], and immunomodulatory potency [76], and presumably transfection efficiency. For example, we showed in our most recent work [77] that LF3000 mediated transgene expression between hBMSCs and hAMSCs, and between donors, varied by up to 10-fold. Similarly, Madeira et al. [78] reported LF2000 mediated transfection efficiency of hBMSCs at passage one to vary between 5 and 20% between donors, and to decrease with increasing passage number. Thus donor variability may explain the differences in reported transfection efficiency by different groups using the same MSC nonviral delivery methods, and direct comparisons of different methods on the same donor(s) are required to truly determine the highest performing nanocarriers. Furthermore, for a nanocarrier to be considered for clinical applications, it must be demonstrated to work on many different donors, which may also require engineering of the nucleic acid cargo, as described next.

### Engineering plasmids and minicircles for nuclear delivery in MSC transfection

In addition to engineering nanocarriers, optimization and incorporation of plasmid sequence elements can be used to enhance nuclear delivery and expression of genes. Plasmid sequence elements (e.g. promoters [79, 80], enhancers, and CpG sites [81, 82]) can determine the rate of transgene transcription in a cell-dependent manner [83], therefore design of plasmid sequences can dramatically affect transgene expression. While many plasmid elements have been studied and optimized in cell lines, these variables still need to be studied in the context of hMSCs. As a first step towards plasmid optimization in hMSCS, we have demonstrated that LF3000 transfection of hBMSCs and hAMSCs with pDNA encoding for a fusion protein of EGFP and luciferase, driven by cytomegalovirus (CMV) promoter, resulted in 10-fold higher transgene expression than transfection with plasmids containing elongation factor 1 α (EF1α) or rous sarcoma virus (RSV) promoters [77], even though the EF1α promoter has been previously shown to outperform the CMV promoter in rat MSCs [84–86]. Comparing these results suggests hMSC physiology may differ significantly from MSCs obtained from other species, and therefore, findings related to transfection efficiency in non-human MSCs must be thoroughly validated in hMSCs.

Aside from promoter selection, another consideration of pDNA design are sequences that may be required for efficient nuclear transport. DNA sequences have been identified that are bound by transcription factors (that present NLS), facilitating import into nuclei, termed DNA targeting sequences (DTS) [87–95]. To investigate the role of DTS-facilitated plasmid transport in hBMSCs and hAMSCs transfected with LF3000, our group has found that removal of the SV40 DTS from pDNA resulted in up to 50% less transgene expression [77] compared to without SV40 DTS removal. Therefore, DTS must be incorporated into pDNA design for efficient MSC transfection.

While plasmid sequence elements can be designed to enhance transfection efficiency in MSCs, plasmid backbone elements like replication of origin and antibiotic resistance genes can trigger intracellular inflammation and transgene silencing [96], potentially limiting transfection efficiency. Therefore, recent transfection studies have investigated minicircle DNA (mcDNA), nucleic acids that are derived from pDNA by recombination that removes bacterial sequences. Narsinh et al. [97] generated mcDNA from parental pDNA by placing a eukaryotic expression cassette containing the desired genes for transfection (without bacterial elements) between sites for ϕC31 integrase recombination. Inducible ϕC31 integrase activity cleaved out the bacterial backbone leaving the remaining eukaryotic cassette as the mcDNA. The mcDNA contained iPSC reprogramming factors OCT4, SOX2, NANOG, and LIN-28, that induced pluripotency in transfected hAMSCs. Electroporated mcDNA increased transfection efficiency 6-fold in transfected hAMSCs compared to pDNA, and after 12 days, transgene mRNA expression was 5-fold higher (resulting in a reprogramming efficiency of about 0.005%). The increase in transfection efficiency and duration of transfection suggest that mcDNA is a promising nonviral vector for MSC gene delivery. However, the additional recombination and purification required to produce mcDNA currently limits widespread use. Therefore, development of optimized pDNA transfection methods for MSCs is still necessary, as well as well as RNA transfection methods that do not require nuclear transport and transcription for expression, as described next.

### Cytoplasmic delivery of mRNA and oligonucleotides in MSC transfection

While there are benefits to plasmid delivery, such as relatively simple manufacture and chemical stability, hMSCs can be more efficiently transfected with mRNAs than with pDNA, presumably by eliminating the need for nuclear transport and transcription of the delivered pDNA. Thus researchers are working to optimize mRNA delivery to MSCs. Lipofection of hMSCs with LF-RNAiMAX complexed with mRNA resulted in 60% transfection efficiency [98], which is significantly higher than typical lipofection efficiencies using pDNA [66–68]. However, while mRNA may mediate higher transfection efficiency, the temporal expression profile of mRNA is shorter in duration than pDNA. For example, nucleofection of MSCs with mRNA has been reported to result in significantly higher early transfection efficiency than with pDNA (80% versus 40%, at day 1, respectively), while pDNA transfection reaches higher efficiency at later time points (25% versus 45%, at day 8) [99]. The shorter duration, but more efficient expression of transgene by mRNA transfection may require repeated dosing, yet may be advantageous in some applications, suggesting that careful selection of nucleic acid cargo is a key transfection design parameter in MSCs.

In addition to mRNA, oligonucleotides can also be transfected for modulation of endogenous gene expression. Small interfering RNA (siRNA) and microRNA (miRNA) are both short RNA oligonucleotides that can inhibit expression of complementary RNAs through binding and inducing cleavage by the RNA-induced silencing complex (RISC) or by inhibiting translation [100]. Like DNA and mRNA, siRNA and miRNA can be delivered via nanocarriers. For example, Benoit et al. [101] developed a di-block co-polymer (pDMAEMA-b-p(DMAEMA-co-PAA-co-BMA)) consisting of an siRNA complexation block (pDMAEMA) and an endosomal escape block (tercopolymer of PAA, BMA, and DMAEMA) for efficient siRNA delivery to hBMSCs. Performance of the di-block co-polymer was compared to the commercial transfection reagent, DharmaFECT, by quantifying hBMSC siRNA uptake, cell viability, and target gene silencing against the housekeeping gene, glyceraldehyde 3-phosphate dehydrogenase (GAPDH). After 24 h, 96% of hBMSCs were siRNA-positive when transfected with the co-polymer, while DharmaFECT transfected at 64% efficiency. There was no significant decrease in viability with co-polymer transfection, while DharmaFECT decreased viability to about 50%. Relative to untreated hBMSCs, the co-polymer decreased GAPDH expression by up to 92%, while DharmaFECT decreased expression by 75% at equivalent siRNA dose. The differences in knockdown and viability in hBMSCs between co-polymer and DharmaFECT mediated siRNA delivery, suggests that choice of nanocarrier is important in oligonucleotide delivery to MSCs. In summary of DNA and RNA delivery, protein expression, and endogenous gene regulation can be achieved with various temporal expression profiles and transfection efficiencies, depending on choice and design of nucleic acid. To complement DNA and RNA delivery, co-delivery of proteins can also be used, as discussed in the next section.

### Co-delivery of proteins and nucleic acids in MSCs ex vivo

Though MSCs can be effectively engineered with nucleic acids, there are applications for which co-delivery of both proteins and nucleic acids may be desirable. For example, towards development of a nanocarrier for co-delivery of an osteogenic transcription factor and pDNA encoding for bone morphogenic protein BMP-2, Park et al. [102] transfected hBMSCs with PEI-coated poly(lactic-co-glycolic acid) (PLGA) nanoparticles, that were loaded with red fluorescent protein (RFP), and coated with pDNA encoding for GFP-tagged BMP2. At 24 h after delivery, 25% of hBMSCs expressed GFP-tagged BMP2, while 33% contained RFP, indicating successful delivery of both protein and pDNA. Co-delivery of proteins and nucleic acids also enables implementation of CRISPR/Cas9 systems for targeted gene modification. Xu et al. [103] used electroporation to co-deliver a Cas9/guide RNA ribonucleoprotein (Cas9/gRNA RNP) with gRNA sequence targeting the beta2-microglobulin (B2M) gene, and a single stranded oligodeoxyribonucleotide (ssODN), to hAMSCs. Co-delivery resulted in B2M expression knock out, from 96% B2M+ in untransfected hAMSCs to 19% B2M+ in transfected hAMSCs, demonstrating successful co-delivery and function of the Cas9/gRNA RNP and ssODN system in hAMSCs. These studies demonstrate successful nonviral co-delivery of proteins and nucleic acids to MSCs for applications that may require the presence of protein prior to, or simultaneously with, nucleic acid expression, expanding the possible methods by which MSC phenotype and genotype can be modulated. However, it is clear that with current delivery methods, transfection efficiency is a primary limitation for applications that seek to make use of nonviral gene transfer to MSCs, therefore innovative approaches for improving nonviral gene delivery are being investigated, as described next.

## Recent approaches to improve nonviral gene delivery to MSCs

Though nucleic acids, carriers, and delivery methods can be optimized for enhanced transfection efficiency in MSCs, culture media additives and culture surface properties can also be optimized to ‘prime’ cells for transfection success, i.e. modulate the cell response to gene transfer in order to enhance transfection efficiency. Next, we present techniques which utilize chemical priming agents (media additives) known to influence transfection efficiency in MSCs, and also briefly discuss the mechanisms by which these additives modulate transfection barriers. Additionally, we present evidence that demonstrates cell culture surface stiffness and protein coatings can influence transfection efficiency in MSCs through physical priming, suggesting further design parameters that must be considered when developing efficient techniques for nucleic acid delivery to these adult stem cells.

### Priming with media additives

#### Glucocorticoids mitigate transfection toxicity to enhance transfection efficiency

After initial identification in a high-throughput screen of over 700 clinically approved compounds to discover priming agents that can enhance transfection in cell lines [104], we have shown that glucocorticoids (Gc) can dramatically enhance transfection in MSCs ex vivo [67]*.* In Kelly et al. [67], we demonstrated in hBMSCs derived from multiple donors, that 100 nM of the Gc dexamethasone (DEX) delivered 0–30 min prior to transfection with three different types of pDNA complexes (formed with either 25 kDa bPEI, LF-2000, or LF-LTX) increased luciferase transgene expression relative to unprimed transfected hBMSCs (3-, 5-, and 10-fold, respectively). In addition to increasing transgene expression, DEX priming of LF-LTX transfection increased hBMSC transfection efficiency about 3-fold, relative to unprimed transfected hBMSCs. We further demonstrated that this DEX-priming effect required binding of the glucocorticoid receptor (GR), by observing that DEX-priming was abrogated when GR binding was inhibited with the GR-antagonist RU486. DEX-primed transfection-increases correlated with rescue of decreased metabolic activity induced by transfection, suggesting that hBMSC transfection toxicity can be ameliorated by DEX priming, through modulation of gene expression by the transcriptional activity of DEX-activated GR [67]. In addition, DEX-primed hMSCs retained their differentiation capacity after transfection, compared to unprimed hMSCs, which exhibited decreased adipogenic and osteogenic differentiation potential after transfection. In Hamann et al. [77], we next investigated the specific mechanisms by which DEX priming enhances transfection of both hBMSCs and hAMSCs, with studies suggesting DEX priming may affect protein synthesis and rescue of transfection-induced apoptosis. In summary, DEX-priming mechanisms suggest that mitigating transfection-induced toxicity can dramatically improve transfection efficiency in MSCs. Therefore, future studies will investigate new candidate priming compounds known to act on relevant stress pathways.

#### Microtubule acetylation and stabilization enhance transfection efficiency

Another transfection priming approach is to improve nuclear localization of pDNA by stabilizing microtubules. Inhibition of cytoplasmic histone deacetylases confers microtubule stability through enrichment of acetyl modifications that increase microtubule flexibility [105]. Dean et al. [106] demonstrated, through histone deacetylase 6 (HDAC6) knockdown, that increased acetylation and improved stability of microtubules results in more efficient pDNA nuclear localization, suggesting HDAC6 inhibition is a potent transfection priming mechanism. Transfection priming with HDAC6 inhibitors has been applied to MSCs to improve transfection. For example, Ho et al. [107] explored priming of transfection to hBMSCs, using 25 kDa linear PEI- primed with the HDAC6 inhibitor, Tubastatin A (10 μM), in combination with DOPE/CHEM, a lipid mixture that facilitates polyplex endosomal escape prior to lysosomal degradation. Relative to unprimed transfected hBMSCs, priming with Tubastatin A and DOPE/CHEM dramatically increased hBMSC transfection efficiency, from 30 to 70%, demonstrating HDAC6 inhibition as a component of an efficient MSC transfection priming strategy. In a similar approach, Dhaliwal et al. [108] transfected mBMSCs with pDNA encoding for luciferase complexed with 25 kDa linear PEI in both 2-D on tissue culture polystyrene (TCPS) and in 3-D culture within RGD (Arg-Gly-Asp) -conjugated hyaluronic acid hydrogels, primed with paclitaxel, which limits microtubule depolymerization. Relative to unprimed transfected mBMSCs, mBMSCs primed with 10 μM paclitaxel 2 h prior to delivery of polyplexes exhibited 8-fold and 35-fold increases in luciferase transgene expression without decreasing viability, in 2-D and 3-D, respectively. These studies reveal the possibility that cytoskeletal modulation can influence transfection efficiency, suggesting further investigations into the interplay between cytoskeletal dynamics and transfection success are needed for improved nucleic acid delivery to MSCs.

### Priming by design of culture surfaces

In addition to microtubule stability, cytoskeletal network tension also plays key roles in rate limiting steps of nonviral gene delivery, through RhoGTPase pathways that modulate the organization of actin stress fibers, which are involved in nanoparticle internalization and endocytic trafficking. [109–114] Actin stress fibers maintain cytoskeletal tension in cell spreading, which is modulated by surface stiffness, surface chemistry, and protein adhesion ligand presentation, and has been demonstrated to correlate with enhanced transfection [115–119]. Therefore, design of these surface properties may be a viable strategy for MSC transfection priming.

In a relatively stiff modulus regime (10 to 670 kPa), Chu et al. [119] transfected mouse D1 BMSCs on fibronectin-coated poly(ethylene glycol) diacrylate hydrogels, using 25 kDa linear PEI complexed with pDNA encoding for bone morphogenic protein BMP-2. BMP-2 transgene expression was significantly increased on 670 kPa versus 10 kPa hydrogels (2-fold increase), corresponding with an observed 6-fold significant increase of polyplex internalization. In comparing soft versus stiff substrates, Modaresi et al. [120] tested delivery of pDNA encoding for vascular endothelial growth factor (VEGF) complexed with LF2000, to hAMSCs cultured on 0.5 or 32 kPa poly(dimethylsiloxane) PDMS surfaces coated with 1% (*w*/*v*) gelatin. VEGF transgene expression was significantly higher (4.5-fold) in hAMSCs cultured on 32 kPa versus 0.5 kPa PDMS, which correlated to a 2.5-fold significant increase in lipoplex internalization, which was shown to be correlated with increased caveolae-mediated endocytosis. Alternatively, within a soft stiffness regime (0.1 to 1.7 kPa), Gojgini et al. [118] demonstrated that mouse BMSCs encapsulated in 3-D hyaluronic acid hydrogels were more spread, and demonstrated increased transfection, at softer stiffnesses. pDNA encoding luciferase was complexed with 25 kDa linear PEI and delivered to BMSCs through incorporation into the hydrogels. As hydrogel stiffness was increased from 0.1 to 1.7 kPa, luciferase transgene expression decreased 5-fold, correlating with increased BMSC spreading and metabolic activity in the softer hydrogels. The authors suggested that decreased migration and spreading due to smaller pore size in the stiffer hydrogels as a potential mechanism that decreased transfection. The results of these studies suggest that transfection efficiency depends on stiffness, and potentially the dimensionality of the culture (i.e. 2-D versus 3-D), and positive or negative correlation of cell spreading and transfection with surface stiffness that depends on stiffness regime [116, 118, 119].

In addition to stiffness tuning, MSC transfection can also be primed by surface or scaffold presentation of proteins. Dhaliwal et al. [121] demonstrated D1 mouse BMSC transfection to vary on TCPS coated with various proteins (vitronectin, collagen I, collagen IV, laminin, fibronectin, and ECMg, a mixture of extracellular matrix (ECM) -derived proteins and proteoglycans). In BMSCs transfected with pDNA encoding for luciferase complexed with 25 kDa linear PEI, transgene expression was increased on coatings of fibronectin, collagen IV, and ECMg (up to 6-, 15-, and 7-fold), decreased on collagen I (up to 10-fold), and not significantly different on laminin or vitronectin, relative to uncoated TCPS. The observed modulation of transgene expression was attributed to coating-dependent differences in cell proliferation, spreading, integrin expression, and polyplex internalization. Therefore, priming of transfection by protein coatings on surfaces may be an approach to improve ex vivo gene delivery to MSCs.

These chemical and physical priming studies suggest that in addition to improvements to nanocarrier design and optimization of electroporation and nucleic acid features, future strategies to optimize nonviral gene delivery to MSCs for clinical applications may utilize the strategies of priming cells both with media additives and culture surface design. With methods and protocols that enable routine transfection in MSCs with high efficiency, clinical applications will be possible, as discussed below.

## Applications of nonviral gene delivery to MSCs

With current nanocarrier and electroporation technology, progress has been made in the development of genetically modified MSC-based cell therapies. We present a broad survey of literature reporting nonviral gene delivery to MSCs, reviewing applications in which MSC transfection conferred additional functionality, illustrating the promise of MSCs for gene therapies that are not limited by the safety concerns associated with viral vectors. Applications of MSC transfection discussed below include tissue engineering, differentiation, reprogramming, promoting survival post-transplantation, directing tissue homing, secretion of therapeutic biomolecules, and cancer therapies.

### Nonviral gene delivery to MSCs for tissue engineering, differentiation, and reprogramming

MSCs have great potential in tissue engineering, particularly for bone and cartilage engineering. A common engineering strategy is to seed MSCs within 3-D scaffolds and provide chemical or physical cues for differentiation into desired tissues. Tissue formation can be enhanced with genetically guided differentiation of MSCs, mediated by nonviral gene delivery [122]. Nonviral gene delivery can also facilitate MSC reprogramming beyond mesodermal lineage. Below, we present research in which MSCs were transfected to engineer bone, cartilage, insulin-secreting cells, vascular tissue, and iPSCs.

#### Gene delivery for MSC differentiation in bone and cartilage tissue engineering

A promising method to deliver recombinant proteins for tissue regeneration, [123, 124] is the use of MSCs, transfected to transiently secrete physiological levels of growth factors to direct specific differentiation and remodeling in target tissue. In an example of bone regeneration, Park et al. [102] co-delivered BMP-2 pDNA with runt-related transcription factor 2 (RUNX2) protein with PEI-coated PLGA nanoparticles to induce osteogenesis of hBMSCs. The combination of BMP-2 expression and transcriptional regulation by RUNX2 was expected to improve osteogenic differentiation over BMP-2 expression or RUNX2 activity alone. In vitro, hBMSCs transfected with both BMP-2 pDNA and RUNX2 protein displayed 20-fold higher osteogenesis-related gene expression than hBMSCs transfected with only BMP-2 pDNA or RUNX2 protein. This trend followed in vivo, resulting in more robust bone regeneration 28 days after subcutaneous injection into nude mice [102]. Similar approaches using MSCs are under study for engineering of articular cartilage to treat defects. For example, in a scaffold-mediated nanocarrier delivery approach, He et al. [125] transfected rat MSCs by seeding cells within gelatin scaffolds containing pDNA encoding transforming growth factor beta-1 (TGFβ-1) complexed with pullulan-spermine, for cartilage regeneration in full-thickness rat cartilage defects. MSCs within scaffolds that delivered TGFβ-1 pDNA were expected to enhance chondrogenesis compared to MSCs within scaffolds that did not deliver TGFβ-1 pDNA. Indeed, proteoglycan and glycosaminoglycan (GAG) histological analysis revealed that scaffolds with transfected MSCs expressing TGFβ-1 induced more cartilage repair than scaffolds with untransfected MSCs [125]. Using transfection prior to scaffold seeding, alternative to the previous scaffold-mediated transfection approach, Bucher et al. directed differentiation of hBMSCs into a phenotype resembling intervertebral disc (IVD) cells by electroporation with pDNA encoding for growth and differentiation factor 5 (GDF5) [126], prior to seeding within alginate hydrogels. Transfected hBMSCs in alginate beads expressed GDF5 for up to 21 days and upregulated chondrogenic markers aggrecan and SOX9, and the discogenic marker KRT19 [126]. When GDF5-expressing hMSCs were injected into an in vitro bovine IVD degeneration model, IVD cartilage regeneration was enhanced, as evidenced by 5-fold increase of GAG to DNA ratio, compared to injection of untransfected hMSCs. These studies demonstrate that MSC differentiation can be successfully guided by nonviral delivery of genes encoding for growth factors.

An alternative to transfection with growth factor genes to direct differentiation is transfection with genes encoding transcription factors that regulate differentiation. Park et al. [127] induced chondrogenesis in hMSCs by delivering pDNA encoding SOX5, SOX6, and SOX9, using PEI on PLGA nanoparticles. As evidenced by staining of collagen II, aggrecan, and cartilage oligomeric matrix protein, hMSCs transfected with the three SOX factors exhibited robust chondrogenesis, while untransfected hMSCs and hMSCs transfected with only individual SOX genes remained undifferentiated [127]. Complementary to regulation of differentiation with transgenic growth factors or transcription factors, oligonucleotide delivery can also direct MSC differentiation through post-transcriptional gene regulation. To guide chondrogenic differentiation by decreasing translation of osteogenic transcription factor RUNX2, Xu et al. [128] delivered complexes of RGD-modified-β-cyclodextrin with RUNX2 siRNA to hMSCs, resulting in significant knockdown of RUNX2 and downregulation of hypertrophy osteogenic marker collagen X in vitro. In subcutaneous hyaluronic acid hydrogels in mice, transfected hMSCs enhanced chondrogenic differentiation, as evidenced by decreased calcification and increased staining of collagen II, GAGs, and proteoglycans. Together, these studies demonstrate successful histologically-verified recapitulation of bone and cartilage generated by MSCs enhanced with nonviral gene delivery in vitro and in animal models and motivates further development of regenerative treatments of bone and cartilage defects utilizing engineered MSCs.

#### Gene delivery for MSC Transdifferentiation

In addition to enhancing tissue formation through mesodermal lineage differentiation, like bone and cartilage, nonviral gene delivery can facilitate MSC differentiation to cell types outside of mesodermal lineage, for example β-cell or endothelial cell transdifferentiation, with uses in treatment of diabetes and vascular tissue engineering, respectively. Through nucleofection of mRNA encoding for PDX-1 prior to chemical induction of β-cell differentiation, Van Pham et al. [129] doubled the efficiency of hUCMSC β-cell differentiation, in comparison to hUCMSCs differentiated by chemical induction alone, achieving up to 8% insulin-positive β-cell phenotype. The enrichment of β-cell differentiation by PDX-1 transfection was functionally verified by two-fold increases in production of insulin and C-peptide in response to glucose [129]. Towards differentiation of MSCs into endothelial cells (ECs), Park et al. [130] induced endothelial differentiation by delivering PLGA nanoparticles loaded with angiogenesis-related peptide (apelin), coated with PEI and pDNA encoding for vascular endothelial growth factor (VEGF) to hMSCs. Transfected hMSCs expressed angiogenic factors, formed tight tubular vessel structures in vitro, and rehabilitated ischemic limbs in mice in vivo by facilitating neovascularization. The successful demonstration of β-cell and EC phenotypes from MSC transdifferentiation utilizing nonviral delivery suggests MSCs can be a cell source for engineering of tissues outside mesodermal lineage, expanding the potential range of MSC clinical applications. Future challenges will include increasing transfection and differentiation efficiency, and demonstration of functional transdifferentiated phenotypes in vivo*.*

#### Gene delivery for reprogramming of MSCs into iPSCs

In addition to being a promising cell type for applications requiring mesodermal- or trans-differentiation, hMSCs are also an attractive source for iPSCs. MSCs have been shown to be induced to pluripotency by integrating viral vectors at 0.2% efficiency, which is 2-fold higher than fibroblast reprogramming efficiency [131]. To bypass the safety drawbacks associated with viral gene delivery discussed earlier, Narsinh et al. [15] reprogrammed hAMSCs into iPSCs by nucleofecting mcDNA encoding for reprogramming factors, Lin28, Nanog, Sox2, and Oct4. Transfection of hAMSCs with mcDNA was 4-fold more efficient than with pDNA, and provided higher transgene expression over a longer period of time. Reprogramming efficiency was only about 0.005% in hAMSCs, which was 10-fold more efficient than reprogramming of differentiated neonatal fibroblasts using the same methods [132]. Alternative to potentially mutagenic viral vectors and inefficient nonviral episomal vectors, reprogramming genes can be integrated at specific sites in the MSC genome, and subsequently removed after iPSC generation, utilizing sequence-specific recombinases. Jia et al. [14] reprogrammed mAMSCs to iPSCs by nucleofecting with pDNA encoding for the reprogramming factors Oct4, Sox2, Klf4, and cMyc, and containing recognition sites for ϕC31 integrase, flanked by loxP sites to allow removal after reprogramming. ϕC31 integrase was expressed from a separate pDNA to integrate the reprogramming cassette into mAMSC genomes. Excision of the reprogramming cassette by Cre recombinase was 50% efficient [14], and reprogramming was achieved at 0.03% efficiency, which is more efficient in comparison to the study above, which achieved reprogramming efficiency of 0.005% using mcDNA [15], though animal source and reprogramming genes differed. These studies demonstrate that MSCs can be successfully reprogrammed into iPSCs via nonviral gene delivery. However nonviral iPSC reprogramming from MSCs remains inefficient, presumably, in part, due to transfection inefficiency. Therefore, methods that improve nonviral gene delivery will enable efficient MSC into iPSC reprogramming via transfection, for clinical-scale applications requiring pluripotent stem cells.

### Nonviral nucleic acid delivery to MSCs for cell therapies

As natural effectors of tissue regeneration that can be accessibly harvested from adult donors, MSCs have been extensively investigated for cell-based therapies in animal models and human trials, for applications ranging from repair of cartilage [133], bone [134], and myocardium [135, 136], to immunosuppression of graft versus host disease (GvHD) and restoration of bone marrow stroma in HSC transplantation [137–139]. Requirements of MSC therapies include cell survival after transplantation, target tissue engraftment, and controlled secretion of therapeutic biomolecules at sufficient levels. As discussed below, ectopic exogenous gene expression has been utilized to enhance and endow hMSCs with these properties towards improving MSC therapeutic efficacy.

#### Promoting transplantation survival

For cell therapies, MSCs must migrate to, and confer their therapeutic effects in, tissue microenvironments of ischemia, inflammation, and oxidative stress, which can result in poor MSC survival. For example, only 7% of MSCs transplanted into infarcted animal myocardium survive, after a few days [140]. For this reason, researchers have investigated ways to enhance survival of transplanted MSCs. One strategy to promote survival is nonviral gene delivery of pro-survival or anti-apoptotic factors to MSCs. For example, Song et al. [141] lipofected rat BMSCs with complexes of LF-PLUS and pDNA encoding for the MSC mitogen, fibroblast growth factor-2 (FGF-2). Compared to untransfected BMSCs, FGF-2 transfection increased BMSC viability 3-fold after 24 h of in vitro hypoxia and serum starvation conditions that simulate transplantation stress, expressing 2-fold higher levels of anti-apoptotic gene Bcl2. When BMSCs were injected into infarcted rat myocardium, FGF-2 transfected BMSCs significantly increased neovascularization after 4 weeks, compared to untransfected BMSCs, presumably, in part, due to FGF-2 induced increase in survival and proliferation after transplantation. Alternatively, MSC survivability under hypoxic conditions could possibly be improved through enzymatic mitigation of heme toxicity. Tang et al. [32] endowed mBMSCs with the ability to inducibly express heme oxygenase-1 (HO-1) in ischemic environments by delivering pDNA encoding for HO-1 complexed with PEI Transferinfection reagent (Bender MedSystems, USA) to mBMSCs. The pDNA was designed with promoter regions driving the HO-1 gene that are recognized by transcription factors GAL4/p65. GAL4/p65, fused with an oxygen degradation domain, was constitutively expressed from the same plasmid, endowing hypoxia-induced HO-1 transcriptional activation to transfected mBMSCS. Transplanted into ischemic mouse myocardiums, transfected mBMSCs exhibited increased survival about 10-fold over control mBMSCs for 7 days, resulting in less myocardial fibrosis and improved hemodynamic heart function [32]. Finally, in an approach alternative to ectopic expression of growth factors or cytoprotective proteins, miRNA delivery can regulate gene expression pathways that promote survival. For example, Xu et al. [142] endowed rat BMSCs with improved ability to survive H_2_O_2_ treatment in vitro by lipofecting BMSCs with miR-20 and LF2000, which increased activity of superoxide dismutase (SOD) and c-Met to reduce oxidative stress and subsequent BMSC apoptosis 2-fold compared to untransfected MSCs, suggesting miR-20 transfection could increase survival and therapeutic effects of MSCs in vivo when exposed to oxidative environments. Overall, through nonviral delivery of miRNA known to regulate oxidative and inflammatory stress responses, along with genes encoding for growth factors and cytoprotective enzymes, MSC transplantation survival can potentially be maximized to enable effective MSC therapies.

#### Directing tissue homing

In addition to transplantation survival, MSCs must reach target tissues in sufficient numbers to realize therapeutic effects. Several groups have utilized nonviral gene delivery to direct MSCs to specific tissues or to enhance the innate tissue homing ability of MSCs, which could improve their therapeutic potential and reduce the MSC dose required for therapeutic effect. Levy et al. [98] enhanced the ability of hBMSCs to selectively invade inflamed tissue by lipofection with LF-RNAiMAX and mRNA encoding for the adhesion ligands PSGL-1 and SLeX, achieving 60% transfection efficiency of both proteins. PSGL-1 and SLeX facilitate cell tethering and rolling on inflamed vascular endothelium. Consequently, 30% more systemically administered transfected hBMSCs localized to inflamed mouse ears than untransfected hBMSCs [98]. In a chemotactic approach, Mun et al. [97] enhanced mBMSC active migration to injury sites by electroporating mcDNA encoding for chemokine receptor type 4 (CXCR4) into mBMSCs, which facilitates migration towards stromal cell-derived factor 1 (SDF1). Transfected mBMSCs maintained transgene expression for up to 7 days [97]. When systemically injected into mice, CXCR4-transfected mBMSCs efficiently homed to full-thickness skin wounds, whereas unmodified mBMSCs accumulated in lungs and were cleared. The directed homing of CXCR4-expressing mBMSCs towards injury resulted in a significant decrease in wound closure time [97]. These studies demonstrate great potential of transfection of MSCs with genes that direct tissue homing to enhance their therapeutic effect and decrease necessary dosing.

### Engineering the MSC Secretome

In addition to engineering MSCs for enhanced survival and tissue-targeting, nonviral gene delivery can enable MSCs to become drug delivery vehicles, secreting transgenic biomolecules that have therapeutic effects, including angiogenic factors, immunomodulatory cytokines, anti-tumorigenic factors, and engineered exosomes, which we discuss below.

#### Growth factors

VEGF is a growth factor that induces endothelial tube formation in angiogenesis. Deveza et al. [143] engineered hAMSCs by delivering pDNA encoding for VEGF complexed with PBAE, resulting in 3-fold more VEGF secretion than unmodified hAMSCs, for up to 8 days. VEGF secreted into media by transfected hAMSCs induced significantly increased angiogenesis by human vein endothelial cells (HUVECs) in vitro relative to media from untransfected hAMSCs. When these VEGF-overexpressing hAMSCs were applied to mouse excision skin wounds, angiogenesis was significantly increased and wound closure time was decreased by 2 days, relative to untransfected hAMSCs [144]. In contrast to transient overexpression of VEGF by MSCs, Cho et al. [145] used a genome engineering approach to achieve sustained expression of VEGF in hUCMSCs. hUCMSCs were transfected with pDNA encoding for an inducible TALEN system that integrated a VEGF sequence into a safe harbor site within the genome. Engineered hUCMSCs secreted 50-fold more VEGF than control hUCMSCs in vitro at 2 weeks after integration of the gene. When engineered hUCMSCs were transplanted into infarcted rat myocardium, heart function was significantly improved by all metrics. Infarct size and fibrosis were also decreased by about 2-fold after 3 weeks, relative to transplant of control hUCMSCs [145]. Thus, transient and sustained VEGF secretion from MSCs has been demonstrated to be effective for angiogenic applications. MSCs can also be transfected to secrete growth factors relevant to other applications. For example, in a neurodegenerative disease application, Dey et al. [146] engineered mBMSCs to secrete about 7-fold more brain derived neurotrophic factor (BDNF) than control mBMSCs by transfection of PEI complexed with pDNA encoding for BDNF. When transplanted into brains of a Huntington’s disease (HD) mouse model, engineered mBMSCs significantly rescued loss of neurons and improved motor function, compared to transplantation of untransfected mBMSCs [146]. To summarize, nonviral gene delivery has been used to achieve both transient and sustained overexpression of growth factors. In animal models, transfected MSCs have produced growth factors that improved wound healing, and enhanced myocardial (VEGF) and neural (BDNF) regeneration, suggesting growth factor secretion from engineered MSCs as a viable strategy for cell therapies.

#### Immunomodulation

MSCs can also be transfected to secrete immunomodulatory factors. Specifically, several studies have investigated MSC secretion of cytokines that reduce inflammation. For example, Levy et al. [98] lipofected hBMSCs with LF-RNAiMAX complexed with mRNA encoding for anti-inflammatory cytokine interleukein-10 (IL-10) to induce IL-10 secretion. Transfected hBMSCs secreted over 10-fold more IL-10 than untransfected hBMSCs, for up to 4 days. Co-culture of transfected hBMSCs with CD4 T cells resulted in about a 2-fold reduction in T cell proliferation in vitro, compared to co-culture with untransfected hBMSCs. When hBMSCs overexpressing IL-10 were administered to mice with inflamed ears, inflamed ear thickness was decreased by about 2-fold, relative to hBMSCs not overexpressing IL-10, indicating enhanced inflammation suppression [98]. Similarly, to reduce injury in a lung inflammation mouse model, Mei et al. [147] electroporated mouse MSCs with pDNA encoding for angiopoietin1 (ANGPT1), a protein that protects against vascular inflammation and promotes EC survival. Transfected MSCs secreted ANGPT1 for 5 days in vitro, and when injected into mouse jugular veins after lipopolysaccharide (LPS)-induced lung injury, transfected MSCs mediated attenuation of inflammation, as evidenced by reduction in neutrophil invasion and inflammatory cytokines, and decreased lung permeability, as evidenced by reductions in IgG and albumin, all compared to injection of untransfected MSCs.

Immunomodulatory factors can also be transfected in MSCs for stable expression. For example, by transposon-mediated integration, Petrakis et al. [148] nucleofected hAMSCs with a pDNA transposon encoding interferon beta-1 (IFNB1) and a separate plasmid encoding the SB100x transposase, resulting in efficient transposition that generated stable expression of IFNB1, demonstrated by 70% of transfected hAMSCs secreting IFNB1 4 weeks later. Though INFB1 is known to upregulate peripheral blood mononuclear cell (PBMC) secretion of immunosuppressive cytokines such as IL-10 and IL-4 [149], PBMC stimulation by the engineered hAMSCs was not tested. However, these studies do demonstrate MSCs can be transfected for transient or stable expression of immunomodulatory factors. Therefore, MSCs can potentially be engineered to decrease inflammation as a facet of MSC-based cell therapies.

#### Cancer therapeutics

Many studies have genetically modified MSCs, which naturally home to tumors [150], to secrete anti-tumorigenic factors, or to express suicide enzymes that cleave pro-drugs, inducing cytotoxicity in tumors. For secretion of an anti-tumorigenic factor, Mangraviti et al. [73] engineered hAMSCs to treat glioblastoma by transfecting with PBAE complexed with pDNA encoding for secreted BMP-4, which significantly suppressed growth of brain tumor initiating cells (BTIC) in vitro. In addition to achieving 75% transfection efficiency and high viability, AMSCs transfected with PBAEs displayed significantly higher motility and invasion in vitro than AMSCs transduced with lentivirus [73]. When engineered hAMSCs were administered intranasally to mice with glioblastoma, survival was prolonged, relative to mice that received control hAMSCs [73]. Another commonly investigated anti-tumorigenic factor for delivery to tumors by MSCs is tumor necrosis factor related apoptosis-inducing ligand (TRAIL). In a specific example, Jiang et al. [151] transfected hAMSCs with PBAE complexed with TRAIL-expressing pDNA, achieving 68% transfection efficiency and about 90% viability, which was 5.5-fold more efficient that LF2000. Transfected hAMSCs were injected into brains of patient-derived tumor xenograft (PDTX) glioblastoma NCr nude mouse model, and migrated to tumor margins. Compared to untransfected hAMSCs, TRAIL-expressing hAMSCs decreased tumor size 2.5-fold and increased survival time. Alternatively to anti-tumorigenic factor secretion, MSCs have also been transfected for suicide gene therapy. For example, Zhang et al. transfected rat BMSCs with spermine-pullulan complexed with pDNA encoding thymidine kinase (TK). BMSCs were injected into a mouse B16F10 pulmonary melanoma metastasis model and migrated to tumor nodules. Upon systemic treatment with pro-drug ganciclovir, TK secreted from transfected BMSCs cleaved ganciclovir to its cytotoxic form within tumors, reducing the number of metastatic lung nodules by 70%, and decreasing lung weight by 30%. In a different novel suicide gene approach that increases radioiodine uptake in tumors, Schug et al. [152] stably transfected hBMSCs with sleeping beauty transposon encoding for sodium iodide symporter (NIS) driven by a TGFβ-1-responsive promoter, to induce expression of NIS when hBMSCs are within tumor stroma that secretes TGFβ-1. Engineered hBMSCs sequestered iodine when stimulated with TGFβ-1 in vitro, and were therefore tested further in vivo*,* injected systemically into mouse liver cancer models. Mice that received radioiodide therapy, exhibited delayed tumor growth and extended survival, relative to mice that did not receive radioiodide therapy [152], suggesting successful tumor-localized, hBMSCs sequestration of radioiodide. To summarize, MSCs can be engineered to secrete anti-tumorigenic factors and to facilitate suicide gene therapy using nonviral gene delivery, with demonstrated effectiveness in animal cancer models that may translate to effective human cancer therapies.

#### Engineering exosome production, targeting, and cargoes

Intercellular transfer of exosomes, which contain organelles, proteins, and RNAs, is thought to be a mechanism by which MSC therapeutic effects are conferred. Isolated MSC exosomes have been utilized to treat pre-clinical models of cardiovascular, neurological, musculoskeletal, and immune system diseases [4]. However, the therapeutic effects of MSC exosomes can be enhanced by nonviral gene delivery. For example, to promote survival and function of transplanted islet cells in a diabetic mouse model, Wen et al. [153] transfected hMSCs with pDNA encoding for siRNA against genes involved in pancreatic islet graft failure, Fas and miR-375. In co-culture with human islet cells in vitro, hMSCs transferred transgenic siRNA to islet cells via exosomes, promoting islet cell survival and rescuing islet cell function decreased by inflammatory cytokines. These transfected hMSCs were then co-transplanted with human pancreatic islets into diabetic mice with humanized immune systems, which resulted in increased islet survival and function, and suppressed islet immune rejection in comparison to islets co-transplanted with untransfected hMSCs [153]. In addition to passive loading of hMSC exosomes with overexpressed oligonucleotides, nonviral gene delivery to increase exosome production and actively load exosomes with transgenic mRNA has also been demonstrated in hMSCs by Kojima et al. [154]. To increase exosome production, hMSCs were electroporated with pDNA encoding for three proteins involved in exosome biogenesis, producing 10-fold more exosomes than untransfected hMSCs, and similarly, through transgenic expression of CD63 fusion proteins with targeting ligands or mRNA-binding petides, hMSC exosomes were engineered to present targeting ligands and load mRNA cargoes [154]. Thus, with efficient nonviral gene delivery, hMSC exosomes can be produced in large quantities, passively or actively loaded with RNA, and targeted with tissue- or cell type-specific ligands, as delivery vehicles for gene therapies.

## Conclusions

MSCs are a promising cell type for allogenic transplantation cell therapies because of their ease of isolation and expansion, multipotent differentiation capacity, and regenerative and immunomodulatory properties. Yet, challenges remain before widespread clinical application of MSC therapies can be realized. Engineering of MSCs through gene delivery approaches could help to overcome barriers to translation of MSC therapies and endow cells with enhanced therapeutic efficacy. A primary concern in the manufacture of genetically modified MSCs is the safety of viral vectors, motivating the development of nonviral vectors. Recent developments in nonviral delivery methods, including nanocarrier technology and plasmid design, in combination with chemical and physical priming of cells during culture ex vivo, may allow for improved nonviral transfection efficiency, enabling scalable translation of genetically engineered MSC therapies for a variety of applications, including guided differentiation and reprogramming, transplantation survival and directed homing, and secretion of therapeutics, potentially bringing effective regenerative medicine to patients.

## Abbreviations

2-D: 2-dimensional
3-D: 3-dimensional
ANGPT1: Angiopoietin 1
B2M: beta2-microglobulin
BDNF: Brain derived neurotrophic factor
BMA: butyl methacrylate
BMP-2: Bone morphogenic protein-2
BMSC: Bone marrow derived MSC
bPEI: Branched PEI
BTIC: Brain tumor initiating cells
Cas9: CRISPR associated protein 9
CD105: Cluster of differentiation 105
CD44: Cluster of differentiation 44
CD63: Cluster of differentiation 63
CD73: Cluster of differentiation 73
CD90: Cluster of differentiation 90
CMV: Cytomegalovirus
CpG: 5’-C-phosphate-G-3’
CRISPR: Clustered regularly interspaced short palindromic repeats
CXCR4: C-X-C chemokine receptor type 4
DEX: Dexamethasone
DNA: Deoxyribonucleic acid
DOPE: 1,2-Dioleoyl-sn-glycero-3-phosphoethanolamine
DTS: DNA targeting sequence
EC: Endothelial cells
ECM: Extracellular matrix
EF1α: elongation factor 1 α
EGFP: Enhanced green fluorescent protein
FDA: Food and Drug Administration
FGF-2: Fibroflast growth factor-2
GAG: Glycosaminoglycan
GAPDH: Glyceraldehyde 3-phosphate dehydrogenase
Gc: Glucocorticoid
GDF5: Growth differentiation factor 5
GFP: Green fluorescent protein
GR: Glucocorticoid receptor
gRNA: Guide RNA
GvHD: Graft versus host disease
H2O2: Hydrogen peroxide
hAMSC: Human adipose derived MSC
hBMSC: Human bone marrow derived MSC
HD: Huntington’s disease
HDAC6: Histone deacetylase 6
hMSC: Human MSC
HO-1: Heme oxygenase-1
HSC: Hematopoietic stem cell
hUMSC: Human umbilical cord MSC
HUVEC: Human vein endothelial cells
IFNB1: interferon beta 1
IgG: immunoglobulin G
IL-10: interleukin-10
IL-4: interleukin-4
iPSC: induced pluripotent stem cell
IVD: intervertebral disc
kDa: kilodalton
kPa: kilopascals
LF: Lipofectamine
LF2000: Lipofectamine 2000
LF3000: Lipofectamine 3000
LF-LTX: Lipofectamine LTX
LF-PLUS: Lipofectamine-Plus
LF-RNAiMAX: Lipofectamine RNAiMAX
LPS: Lipopolysaccharide
mAMSC: mouse AMSC
mBMSC: mouse BMSCs
mcDNA: minicircle DNA
miRNA: micro RNA
mRNA: messenger RNA
MSC: Mesenchymal stem cell
nHA: nano-hydroxyapatite
NIS: sodium iodide symporter
NLS: Nuclear localization sequence
nm: nanometer
nM: nanomolar
PAA: Propylacrylic acid
PAMAM: Poly(amidoamine)
PBAE: Poly(β-amino-esters)
PBMC: Peripheral blood mononuclear cells
pDMAEMA: poly(dimethylaminoethyl methacrylate)
PDMS: Polydimethylsiloxane
pDNA: plasmid DNA
PDTX: Patient-derived tumor xenograft
PEI: Polyethylenimine
pH: decimal co-logarithm of hydrogen
PLGA: Poly(lactic-co-glycolic acid)
RALA: Repeating arginine-alanine-leucine-alanine
RFP: Red fluorescent protein
RGD: Arg-Gly-Asp
RISC: RNA-induced silencing complex
RNA: Ribonucleic acid
RSV: Rous sarcoma virus
RUNX2: Runt-related transcription factor 2
SDF1: Stromal cell-derived factor 1
siRNA: small interfering RNA
SOD: Superoxide dismutase
ssODN: Single stranded oligodeoxynucleotide
SV40: Simian virus 40
TALEN: Transcription activator-like effector nuclease
TCPS: Tissue culture polystyrene
TGFβ-1: Transforming growth factor β-1
TK: Thymidine kinase
TRAIL: Tumor necrosis factor related apoptosis inducing ligand
VEGF: Vascular endothelial growth factor
*w*/*v*: weight/volume

## References

[1] Wei X, Yang X, Han Z-P, Qu F-F, Shao L, Shi Y-F (2013). Mesenchymal stem cells: a new trend for cell therapy. Acta Pharmacol Sin.

[2] Dominici M, Le Blanc K, Mueller I, Slaper-Cortenbach I, Marini F, Krause D (2006). Minimal criteria for defining multipotent mesenchymal stromal cells. The International Society for Cellular Therapy position statement. Cytotherapy.

[3] Chamberlain G, Fox J, Ashton B, Middleton J (2007). Concise review: mesenchymal stem cells: their phenotype, differentiation capacity, immunological features, and potential for homing. Stem Cells.

[4] Phan J, Kumar P, Hao D, Gao K, Farmer D, Wang A (2018). Engineering mesenchymal stem cells to improve their exosome efficacy and yield for cell-free therapy. J Extracell Vesicles.

[5] Wada N, Gronthos S, Bartold PM (2013). Immunomodulatory effects of stem cells. Periodontol.

[6] Glennie S, Soeiro I, Dyson PJ, Lam EWF, Dazzi F (2004). Bone marrow mesenchymal stem cells induce division arrest anergy of activated T cells. Blood.

[7] da Silva ML, Caplan AI, Nardi NB (2008). In search of the in vivo identity of mesenchymal stem cells. Stem Cells.

[8] Maxson S, Lopez EA, Yoo D, Danilkovitch-Miagkova A, Leroux MA (2012). Concise review: role of mesenchymal stem cells in wound repair. Stem Cells Transl Med.

[9] Singer NG, Caplan AI (2011). Mesenchymal stem cells: mechanisms of inflammation. Annu Rev Pathol.

[10] D’souza N, Rossignoli F, Golinelli G, Grisendi G, Spano C, Candini O (2015). Mesenchymal stem/stromal cells as a delivery platform in cell and gene therapies. BMC Med.

[11] Goepfert C, Slobodianski A, Schilling A, Adamietz P, Pörtner R (2010). Cartilage engineering from mesenchymal stem cells. Bioreactor Systems for Tissue Engineering II: Springer.

[12] Gafni Y, Turgeman G, Liebergal M, Pelled G, Gazit Z, Gazit D (2004). Stem cells as vehicles for orthopedic gene therapy. Gene Ther.

[13] Ankrum JA, Ong JF, Karp JM (2014). Mesenchymal stem cells: immune evasive, not immune privileged. Nat Biotechnol.

[14] Karow M, Chavez CL, Farruggio AP, Geisinger JM, Keravala A, Jung WE (2011). Site-specific recombinase strategy to create induced pluripotent stem cells efficiently with plasmid DNA. Stem Cells.

[15] Narsinh KH, Jia F, Robbins RC, Kay MA, Longaker MT, Wu JC (2011). Generation of adult human induced pluripotent stem cells using nonviral minicircle DNA vectors. Nat Protoc.

[16] Oggu GS, Sasikumar S, Reddy N, Ella KKR, Rao CM, Bokara KK (2017). Gene delivery approaches for mesenchymal stem cell therapy: strategies to increase efficiency and specificity. Stem Cell Rev Rep.

[17] Galipeau J, Sensébé L (2018). Mesenchymal stromal cells: clinical challenges and therapeutic opportunities. Cell Stem Cell.

[18] Zhang J, Huang X, Wang H, Liu X, Zhang T, Wang Y (2015). The challenges and promises of allogeneic mesenchymal stem cells for use as a cell-based therapy. Stem Cell Rev Rep.

[19] Lee S, Choi E, Cha M-J, Hwang K-C. Cell adhesion and long-term survival of transplanted mesenchymal stem cells: a prerequisite for cell therapy. Oxidative Med Cell Longev. 2015;2015:632902. 10.1155/2015/632902 PMC4333334 25722795

[20] Schrepfer S, Deuse T, Reichenspurner H, Fischbein MP, Robbins RC, Pelletier M. Stem cell transplantation: the lung barrier. Transplant Proc; 2007;39:573–76. 10.1016/j.transproceed.2006.12.019 17362785

[21] Yang Y-HK, Ogando CR, See CW, Chang T-Y, Barabino GA (2018). Changes in phenotype and differentiation potential of human mesenchymal stem cells aging in vitro. Stem Cell Res Ther.

[22] Fekete N, Rojewski MT, Lotfi R, Schrezenmeier H (2013). Essential components for ex vivo proliferation of mesenchymal stromal cells. Tissue Engineering Part C: Methods.

[23] Oh J-E, Eom YW (2016). Maintenance of proliferation and adipogenic differentiation by fibroblast growth factor-2 and dexamethasone through expression of hepatocyte growth factor in bone marrow-derived mesenchymal stem cells. Biomed Sci Lett.

[24] Eom YW, Oh J-E, Lee JI, Baik SK, Rhee K-J, Shin HC (2014). The role of growth factors in maintenance of stemness in bone marrow-derived mesenchymal stem cells. Biochem Biophys Res Commun.

[25] Li Y, Guo G, Li L, Chen F, Bao J, Shi Y-J (2015). Three-dimensional spheroid culture of human umbilical cord mesenchymal stem cells promotes cell yield and stemness maintenance. Cell Tissue Res.

[26] Rakian R, Block TJ, Johnson SM, Marinkovic M, Wu J, Dai Q (2015). Native extracellular matrix preserves mesenchymal stem cell “stemness” and differentiation potential under serum-free culture conditions. Stem Cell Res Ther.

[27] Heo SJ, Szczesny SE, Kim DH, Saleh KS, Mauck RL (2018). Expansion of mesenchymal stem cells on electrospun scaffolds maintains stemness, mechano-responsivity, and differentiation potential. J Orthop Res.

[28] Liu L, DiGirolamo CM, Navarro PA, Blasco MA, Keefe DL (2004). Telomerase deficiency impairs differentiation of mesenchymal stem cells. Exp Cell Res.

[29] Sachs PC, Francis MP, Zhao M, Brumelle J, Rao RR, Elmore LW (2012). Defining essential stem cell characteristics in adipose-derived stromal cells extracted from distinct anatomical sites. Cell Tissue Res.

[30] Piper SL, Wang M, Yamamoto A, Malek F, Luu A, Kuo AC (2012). Inducible immortality in hTERT-human mesenchymal stem cells. J Orthop Res.

[31] Siska EK, Weisman I, Romano J, Ivics Z, Izsvák Z, Barkai U (2017). Generation of an immortalized mesenchymal stem cell line producing a secreted biosensor protein for glucose monitoring. PLoS One.

[32] Tang YL, Tang Y, Zhang YC, Qian K, Shen L, Phillips MI (2005). Improved graft mesenchymal stem cell survival in ischemic heart with a hypoxia-regulated heme oxygenase-1 vector. J Am Coll Cardiol.

[33] Baldari S, Di Rocco G, Piccoli M, Pozzobon M, Muraca M, Toietta G (2017). Challenges and strategies for improving the regenerative effects of mesenchymal stromal cell-based therapies. Int J Mol Sci.

[34] Cheng Z, Ou L, Zhou X, Li F, Jia X, Zhang Y (2008). Targeted migration of mesenchymal stem cells modified with CXCR4 gene to infarcted myocardium improves cardiac performance. Mol Ther.

[35] Huang J, Zhang Z, Guo J, Ni A, Deb A, Zhang L (2010). Genetic modification of mesenchymal stem cells overexpressing CCR1 increases cell viability, migration, engraftment, and capillary density in the injured myocardium. Circ Res.

[36] Kumar S, Ponnazhagan S (2007). Bone homing of mesenchymal stem cells by ectopic α4 integrin expression. FASEB J.

[37] Tsuchiya H, Kitoh H, Sugiura F, Ishiguro N (2003). Chondrogenesis enhanced by overexpression of sox9 gene in mouse bone marrow-derived mesenchymal stem cells. Biochem Biophys Res Commun.

[38] Alberton P, Popov C, Prägert M, Kohler J, Shukunami C, Schieker M (2011). Conversion of human bone marrow-derived mesenchymal stem cells into tendon progenitor cells by ectopic expression of scleraxis. Stem Cells Dev.

[39] Beegle JR, Magner NL, Kalomoiris S, Harding A, Zhou P, Nacey C, et al. Preclinical evaluation of mesenchymal stem cells overexpressing VEGF to treat critical limb ischemia. Mol Ther Methods Clin Dev. 2016;3:16053. 10.1038/mtm.2016.53 PMC5003097 27610394

[40] Pollock K, Dahlenburg H, Nelson H, Fink KD, Cary W, Hendrix K (2016). Human mesenchymal stem cells genetically engineered to overexpress brain-derived neurotrophic factor improve outcomes in Huntington's disease mouse models. Mol Ther.

[41] Choi JJ, Yoo SA, Park SJ, Kang YJ, Kim WU, Oh IH (2008). Mesenchymal stem cells overexpressing interleukin-10 attenuate collagen-induced arthritis in mice. Clin Exp Immunol.

[42] Nakajima M, Nito C, Sowa K, Suda S, Nishiyama Y, Nakamura-Takahashi A (2017). Mesenchymal stem cells overexpressing interleukin-10 promote neuroprotection in experimental acute ischemic stroke. Mol Ther Methods Clin Dev.

[43] Yu B, Kim HW, Gong M, Wang J, Millard RW, Wang Y (2015). Exosomes secreted from GATA-4 overexpressing mesenchymal stem cells serve as a reservoir of anti-apoptotic microRNAs for cardioprotection. Int J Cardiol.

[44] Baglio SR, Rooijers K, Koppers-Lalic D, Verweij FJ, Lanzón MP, Zini N (2015). Human bone marrow-and adipose-mesenchymal stem cells secrete exosomes enriched in distinctive miRNA and tRNA species. Stem Cell Res Ther.

[45] Giacca M, Zacchigna S (2012). Virus-mediated gene delivery for human gene therapy. J Control Release.

[46] Barkholt L, Flory E, Jekerle V, Lucas-Samuel S, Ahnert P, Bisset L (2013). Risk of tumorigenicity in mesenchymal stromal cell–based therapies—bridging scientific observations and regulatory viewpoints. Cytotherapy.

[47] Wuchter P, Bieback K, Schrezenmeier H, Bornhäuser M, Müller LP, Bönig H (2015). Standardization of good manufacturing practice–compliant production of bone marrow–derived human mesenchymal stromal cells for immunotherapeutic applications. Cytotherapy.

[48] Chan JL, Tang KC, Patel AP, Bonilla LM, Pierobon N, Ponzio NM (2006). Antigen-presenting property of mesenchymal stem cells occurs during a narrow window at low levels of interferon-γ. Blood.

[49] Dewey R, Morrissey G, Cowsill C, Stone D, Bolognani F, Dodd N (1999). Chronic brain inflammation and persistent herpes simplex virus 1 thymidine kinase expression in survivors of syngeneic glioma treated by adenovirus-mediated gene therapy: implications for clinical trials. Nat Med.

[50] Yin H, Kanasty RL, Eltoukhy AA, Vegas AJ, Dorkin JR, Anderson DG (2014). Non-viral vectors for gene-based therapy. Nat Rev Genet.

[51] Nayerossadat N, Maedeh T, Ali PA (2012). Viral and nonviral delivery systems for gene delivery. Adv Biomed Res.

[52] Dean D, Strong D, Zimmer W (2005). Nuclear entry of nonviral vectors. Gene Ther.

[53] Tsulaia TV, Prokopishyn NL, Yao A, Carsrud NV, Carou MC, Brown DB (2003). Glass needle-mediated microinjection of macromolecules and transgenes into primary human mesenchymal stem cells. J Biomed Sci.

[54] Han S-W, Nakamura C, Kotobuki N, Obataya I, Ohgushi H, Nagamune T (2008). High-efficiency DNA injection into a single human mesenchymal stem cell using a nanoneedle and atomic force microscopy. Nanomed Nanotechnol Biol Med.

[55] Stewart MP, Langer R, Jensen KF (2018). Intracellular delivery by membrane disruption: mechanisms, strategies, and concepts. Chem Rev.

[56] Wu M, Yuan F (2011). Membrane binding of plasmid DNA and endocytic pathways are involved in electrotransfection of mammalian cells. PLoS One.

[57] Wang W, Xu X, Li Z, Lendlein A, Ma N (2014). Genetic engineering of mesenchymal stem cells by non-viral gene delivery. Clin Hemorheol Microcirc.

[58] Cervia LD, Chang C-C, Wang L, Mao M, Yuan F (2018). Enhancing Electrotransfection efficiency through improvement in nuclear entry of plasmid DNA. Mol Ther Nucleic Acids.

[59] Gresch O, Engel FB, Nesic D, Tran TT, England HM, Hickman ES (2004). New non-viral method for gene transfer into primary cells. Methods.

[60] Nakashima S, Matsuyama Y, Nitta A, Sakai Y, Ishiguro N, editors. Highly efficient transfection of human marrow stromal cells by nucleofection. Transplant Proc. 2005;37:2290–92. 10.1016/j.transproceed.2005.03.047 15964401

[61] Aslan H, Zilberman Y, Arbeli V, Sheyn D, Matan Y, Liebergall M (2006). Nucleofection-based ex vivo nonviral gene delivery to human stem cells as a platform for tissue regeneration. Tissue Eng.

[62] Kim JA, Cho K, Shin MS, Lee WG, Jung N, Chung C (2008). A novel electroporation method using a capillary and wire-type electrode. Biosens Bioelectron.

[63] Lim JY, Park SH, Jeong CH, Oh JH, Kim SM, Ryu CH (2010). Microporation is a valuable transfection method for efficient gene delivery into human umbilical cord blood-derived mesenchymal stem cells. BMC Biotechnol.

[64] Xiang S, Tong H, Shi Q, Fernandes JC, Jin T, Dai K (2012). Uptake mechanisms of non-viral gene delivery. J Control Release.

[65] Santos JL, Pandita D, Rodrigues J, Pego AP, Granja PL, Tomás H (2011). Non-viral gene delivery to mesenchymal stem cells: methods, strategies and application in bone tissue engineering and regeneration. Curr Gene Ther.

[66] Hoare M, Greiser U, Schu S, Mashayekhi K, Aydogan E, Murphy M (2010). Enhanced lipoplex-mediated gene expression in mesenchymal stem cells using reiterated nuclear localization sequence peptides. J Gene Med..

[67] Kelly AM, Plautz SA, Zempleni J, Pannier AK (2016). Glucocorticoid cell priming enhances transfection outcomes in adult human mesenchymal stem cells. Mol Ther.

[68] de Carvalho TG, Pellenz FM, Laureano A, da Rocha Silla LM, Giugliani R, Baldo G (2018). A simple protocol for transfecting human mesenchymal stem cells. Biotechnol Lett.

[69] Gonzalez-Fernandez T, Sathy B, Hobbs C, Cunniffe G, McCarthy H, Dunne N (2017). Mesenchymal stem cell fate following non-viral gene transfection strongly depends on the choice of delivery vector. Acta Biomater.

[70] Saraf A, Hacker MC, Sitharaman B, Grande-Allen KJ, Barry MA, Mikos AG (2008). Synthesis and conformational evaluation of a novel gene delivery vector for human mesenchymal stem cells. Biomacromolecules.

[71] Santos JL, Pandita D, Rodrigues J, Pêgo AP, Granja PL, Balian G (2010). Receptor-mediated gene delivery using PAMAM dendrimers conjugated with peptides recognized by mesenchymal stem cells. Mol Pharm.

[72] Badding MA, Lapek JD, Friedman AE, Dean DA (2013). Proteomic and functional analyses of protein-DNA complexes during gene transfer. Mol Ther.

[73] Mangraviti A, Tzeng SY, Gullotti D, Kozielski KL, Kim JE, Seng M (2016). Non-virally engineered human adipose mesenchymal stem cells produce BMP4, target brain tumors, and extend survival. Biomaterials.

[74] Qadan MA, Piuzzi NS, Boehm C, Bova W, Moos M, Midura RJ (2018). Variation in primary and culture-expanded cells derived from connective tissue progenitors in human bone marrow space, bone trabecular surface and adipose tissue. Cytotherapy.

[75] Mohamed-Ahmed S, Fristad I, Lie SA, Suliman S, Mustafa K, Vindenes H (2018). Adipose-derived and bone marrow mesenchymal stem cells: a donor-matched comparison. Stem Cell Res Ther.

[76] Ketterl N, Brachtl G, Schuh C, Bieback K, Schallmoser K, Reinisch A (2015). A robust potency assay highlights significant donor variation of human mesenchymal stem/progenitor cell immune modulatory capacity and extended radio-resistance. Stem Cell Res Ther.

[77] Hamann A, Broad K, Nguyen A, Pannier AK. Mechanisms of unprimed and dexamethasone-primed nonviral gene delivery to human mesenchymal stem cells. Biotechnol Bioeng. 2019;116:427–43. 10.1002/bit.26870 PMC6322959 30450542

[78] Madeira C, Mendes R, Ribeiro S, Boura J, Aires-Barros M, da Silva C, et al. Nonviral gene delivery to mesenchymal stem cells using cationic liposomes for gene and cell therapy. Biomed Res Int. 2010;2010:735349. 10.1155/2010/735349 PMC2896879 20625411

[79] Lebbink RJ, Lowe M, Chan T, Khine H, Wang X, McManus MT (2011). Polymerase II promoter strength determines efficacy of microRNA adapted shRNAs. PLoS One.

[80] Wright E, Bain M, Teague L, Murphy J, Sinclair J (2005). Ets-2 repressor factor recruits histone deacetylase to silence human cytomegalovirus immediate-early gene expression in non-permissive cells. J Gen Virol.

[81] Prösch S, Stein J, Staak K, Liebenthal C, Volk H-D, Krüger DH (1996). Inactivation of the very strong HCMV immediate early promoter by DNA CpG methylation in vitro. Biol Chem.

[82] Hong K, Sherley J, Lauffenburger DA (2001). Methylation of episomal plasmids as a barrier to transient gene expression via a synthetic delivery vector. Biomol Eng.

[83] Li S, MacLaughlin F, Fewell J, Gondo M, Wang J, Nicol F (2001). Muscle-specific enhancement of gene expression by incorporation of SV40 enhancer in the expression plasmid. Gene Ther.

[84] Ferreira E, Potier E, Vaudin P, Oudina K, Bensidhoum M, Logeart-Avramoglou D, et al. Sustained and promoter dependent bone morphogenetic protein expression by rat mesenchymal stem cells after BMP-2 transgene electrotransfer. Eur Cell Mater. 2012;24:18–28. 10.22203/ecm.v024a02 22777950

[85] McGinley L, McMahon J, Strappe P, Barry F, Murphy M, O'Toole D (2011). Lentiviral vector mediated modification of mesenchymal stem cells & enhanced survival in an in vitro model of ischaemia. Stem Cell Res Ther.

[86] Qin JY, Zhang L, Clift KL, Hulur I, Xiang AP, Ren B-Z (2010). Systematic comparison of constitutive promoters and the doxycycline-inducible promoter. PLoS One.

[87] Young JL, Benoit JN, Dean DA (2003). Effect of a DNA nuclear targeting sequence on gene transfer and expression of plasmids in the intact vasculature. Gene Ther.

[88] Cramer F, Christensen C, Poulsen T, Badding M, Dean D, Poulsen H (1910). Insertion of a nuclear factor kappa B DNA nuclear-targeting sequence potentiates suicide gene therapy efficacy in lung cancer cell lines. Cancer Gene Ther.

[89] Breuzard G, Tertil M, Goncalves C, Cheradame H, Geguan P, Pichon C (2008). Nuclear delivery of NFκB-assisted DNA/polymer complexes: plasmid DNA quantitation by confocal laser scanning microscopy and evidence of nuclear polyplexes by FRET imaging. Nucleic Acids Res.

[90] Wilson GL, Dean BS, Wang G, Dean DA (1999). Nuclear import of plasmid DNA in digitonin-permeabilized cells requires both cytoplasmic factors and specific DNA sequences. Biol Chem.

[91] Gonçalves C, Ardourel MY, Decoville M, Breuzard G, Midoux P, Hartmann B (2009). An optimized extended DNA kappa B site that enhances plasmid DNA nuclear import and gene expression. J Gene Med..

[92] Miller AM, Dean DA (2009). Tissue-specific and transcription factor-mediated nuclear entry of DNA. Adv Drug Deliv Rev.

[93] Badding MA, Vaughan EE, Dean DA (2012). Transcription factor plasmid binding modulates microtubule interactions and intracellular trafficking during gene transfer. Gene Ther.

[94] Van Gaal EV, Oosting RS, van Eijk R, Bakowska M, Feyen D, Kok RJ (2011). DNA nuclear targeting sequences for non-viral gene delivery. Pharm Res.

[95] Dean DA (1997). Import of plasmid DNA into the nucleus is sequence specific. Exp Cell Res.

[96] Hardee CL, Arévalo-Soliz LM, Hornstein BD, Zechiedrich L (2017). Advances in non-viral DNA vectors for gene therapy. Genes.

[97] Mun J-Y, Shin KK, Kwon O, Lim YT, Oh D-B (2016). Minicircle microporation-based non-viral gene delivery improved the targeting of mesenchymal stem cells to an injury site. Biomaterials.

[98] Levy O, Zhao W, Mortensen LJ, LeBlanc S, Tsang K, Fu M, et al. mRNA-engineered mesenchymal stem cells for targeted delivery of interleukin-10 to sites of inflammation. Blood. 2013;122:e23–32. 10.1182/blood-2013-04-495119 PMC3790516 23980067

[99] Wiehe JM, Ponsaerts P, Rojewski MT, Homann JM, Greiner J, Kronawitter D (2007). mRNA-mediated gene delivery into human progenitor cells promotes highly efficient protein expression. J Cell Mol Med.

[100] Gu S, Kay MA. How do miRNAs mediate translational repression? Silence. 2010;1:11. 10.1186/1758-907X-1-11 PMC2881910 20459656

[101] Benoit DS, Boutin ME (2012). Controlling mesenchymal stem cell gene expression using polymer-mediated delivery of siRNA. Biomacromolecules.

[102] Park JS, Yi SW, Kim HJ, Kim SM, Park K-H (2016). Regulation of cell signaling factors using PLGA nanoparticles coated/loaded with genes and proteins for osteogenesis of human mesenchymal stem cells. ACS Appl Mater Interfaces.

[103] Xu X, Gao D, Wang P, Chen J, Ruan J, Xu J (2018). Efficient homology-directed gene editing by CRISPR/Cas9 in human stem and primary cells using tube electroporation. Sci Rep.

[104] Nguyen A, Beyersdorf J, Riethoven JJ, Pannier AK (2016). High-throughput screening of clinically approved drugs that prime polyethylenimine transfection reveals modulation of mitochondria dysfunction response improves gene transfer efficiencies. Bioeng Transl Med.

[105] Janke C, Montagnac G (2017). Causes and consequences of microtubule acetylation. Curr Biol.

[106] Vaughan EE, Geiger RC, Miller AM, Loh-Marley PL, Suzuki T, Miyata N (2008). Microtubule acetylation through HDAC6 inhibition results in increased transfection efficiency. Mol Ther.

[107] Ho YK, Zhou LH, Tam KC, Too HP (2017). Enhanced non-viral gene delivery by coordinated endosomal release and inhibition of β-tubulin deactylase. Nucleic Acids Res.

[108] Dhaliwal A, Oshita V, Segura T (2013). Transfection in the third dimension. Integr Biol.

[109] Adler AF, Leong KW (2010). Emerging links between surface nanotechnology and endocytosis: impact on nonviral gene delivery. Nano Today.

[110] Dhaliwal A, Maldonado M, Lin C, Segura T (2012). Cellular cytoskeleton dynamics modulates non-viral gene delivery through RhoGTPases. PLoS One.

[111] Rosazza C, Escoffre J-M, Zumbusch A, Rols M-P (2011). The actin cytoskeleton has an active role in the electrotransfer of plasmid DNA in mammalian cells. Mol Ther.

[112] Kopatz I, Remy JS, Behr JP (2004). A model for non-viral gene delivery: through syndecan adhesion molecules and powered by actin. J Gene Med..

[113] Dauty E, Verkman A (2005). Actin cytoskeleton as the principal determinant of size-dependent DNA mobility in cytoplasm a new barrier for non-viral gene delivery. Biol Chem.

[114] Bausinger R, von Gersdorff K, Braeckmans K, Ogris M, Wagner E, Bräuchle C (2006). The transport of nanosized gene carriers unraveled by live-cell imaging. Angew Chem.

[115] Kasputis T, Pannier AK (2012). The role of surface chemistry-induced cell characteristics on nonviral gene delivery to mouse fibroblasts. J Biol Eng.

[116] Kong HJ, Liu J, Riddle K, Matsumoto T, Leach K, Mooney DJ (2005). Non-viral gene delivery regulated by stiffness of cell adhesion substrates. Nat Mater.

[117] Kong HJ, Hsiong S, Mooney DJ (2007). Nanoscale cell adhesion ligand presentation regulates nonviral gene delivery and expression. Nano Lett.

[118] Gojgini S, Tokatlian T, Segura T (2011). Utilizing cell–matrix interactions to modulate gene transfer to stem cells inside hyaluronic acid hydrogels. Mol Pharm.

[119] Chu C, Kong H (2012). Interplay of cell adhesion matrix stiffness and cell type for non-viral gene delivery. Acta Biomater.

[120] Modaresi S, Pacelli S, Whitlow J, Paul A (2018). Deciphering the role of substrate stiffness in enhancing the internalization efficiency of plasmid DNA in stem cells using lipid-based nanocarriers. Nanoscale.

[121] Dhaliwal A, Lam J, Maldonado M, Lin C, Segura T (2012). Extracellular matrix modulates non-viral gene transfer to mouse mesenchymal stem cells. Soft Matter.

[122] Raisin S, Belamie E, Morille M (2016). Non-viral gene activated matrices for mesenchymal stem cells based tissue engineering of bone and cartilage. Biomaterials.

[123] Woo EJ (2012). Adverse events reported after the use of recombinant human bone morphogenetic protein 2. J Oral Maxillofac Surg.

[124] Tannoury CA, An HS (2014). Complications with the use of bone morphogenetic protein 2 (BMP-2) in spine surgery. Spine J.

[125] He CX, Zhang TY, Miao PH, Hu ZJ, Han M, Tabata Y (2012). TGF-β1 gene-engineered mesenchymal stem cells induce rat cartilage regeneration using nonviral gene vector. Biotechnol Appl Biochem.

[126] Bucher C, Gazdhar A, Benneker LM, Geiser T, Gantenbein-Ritter B. Nonviral gene delivery of growth and differentiation factor 5 to human mesenchymal stem cells injected into a 3D bovine intervertebral disc organ culture system. Stem Cells Int. 2013;2013:326828. 10.1155/2013/326828 PMC3885261 24454406

[127] Park JS, Yi SW, Kim HJ, Kim SM, Kim J-H, Park K-H (2017). Construction of PLGA nanoparticles coated with Polycistronic SOX5, SOX6, and SOX9 genes for Chondrogenesis of human mesenchymal stem cells. ACS Appl Mater Interfaces.

[128] Xu J, Li J, Lin S, Wu T, Huang H, Zhang K (2016). Nanocarrier-mediated Codelivery of small molecular drugs and siRNA to enhance Chondrogenic differentiation and suppress hypertrophy of human mesenchymal stem cells. Adv Funct Mater.

[129] Van Pham P, Nguyen PT-M, Nguyen AT-Q, Pham VM, Bui AN-T, Dang LT-T (2014). Improved differentiation of umbilical cord blood-derived mesenchymal stem cells into insulin-producing cells by PDX-1 mRNA transfection. Differentiation.

[130] Park JS, Yang HN, Yi SW, Kim J-H, Park K-H (2016). Neoangiogenesis of human mesenchymal stem cells transfected with peptide-loaded and gene-coated PLGA nanoparticles. Biomaterials.

[131] Streckfuss-Bömeke K, Wolf F, Azizian A, Stauske M, Tiburcy M, Wagner S (2012). Comparative study of human-induced pluripotent stem cells derived from bone marrow cells, hair keratinocytes, and skin fibroblasts. Eur Heart J.

[132] Jia F, Wilson KD, Sun N, Gupta DM, Huang M, Li Z (2010). A nonviral minicircle vector for deriving human iPS cells. Nat Methods.

[133] Haleem AM, Singergy AAE, Sabry D, Atta HM, Rashed LA, Chu CR (2010). The clinical use of human culture–expanded autologous bone marrow mesenchymal stem cells transplanted on platelet-rich fibrin glue in the treatment of articular cartilage defects: a pilot study and preliminary results. Cartilage.

[134] Horwitz EM, Gordon PL, Koo WK, Marx JC, Neel MD, McNall RY (2002). Isolated allogeneic bone marrow-derived mesenchymal cells engraft and stimulate growth in children with osteogenesis imperfecta: implications for cell therapy of bone. PNAS.

[135] Hare JM, Traverse JH, Henry TD, Dib N, Strumpf RK, Schulman SP (2009). A randomized, double-blind, placebo-controlled, dose-escalation study of intravenous adult human mesenchymal stem cells (prochymal) after acute myocardial infarction. J Am Coll Cardiol.

[136] Amado LC, Saliaris AP, Schuleri KH, John MS, Xie J-S, Cattaneo S (2005). Cardiac repair with intramyocardial injection of allogeneic mesenchymal stem cells after myocardial infarction. PNAS.

[137] Servais S, Baron F, Lechanteur C, Seidel L, Selleslag D, Maertens J (2018). Infusion of bone marrow derived multipotent mesenchymal stromal cells for the treatment of steroid-refractory acute graft-versus-host disease: a multicenter prospective study. Oncotarget.

[138] Castello LM, Leone M, Adamini A, Castiglia S, Mareschi K, Ferrero I (2018). Analysis of mesenchymal stromal cell engraftment after allogeneic HSCT in pediatric patients: a large multicenter study. J Pediatr Hematol Oncol.

[139] Wang D, Zhang H, Liang J, Wang H, Hua B, Feng X (2018). A long-term follow-up study of allogeneic mesenchymal stem/stromal cell transplantation in patients with drug-resistant systemic lupus erythematosus. Stem Cell Rep.

[140] Li L, Chen X, Wang WE, Zeng C. How to improve the survival of transplanted mesenchymal stem cell in ischemic heart? Stem Cells Int. 2016;2016:9682757. 10.1155/2016/9682757 PMC4670674 26681958

[141] Song H, Kwon K, Lim S, Kang S-M, Ko Y-G, Xu Z, et al. Transfection of mesenchymal stem cells with the FGF-2 gene improves their survival under hypoxic conditions. Mol Cells. 2005;19:402–7. 15995358

[142] Xu J, Huang Z, Lin L, Fu M, Gao Y, Shen Y (2014). miR-210 over-expression enhances mesenchymal stem cell survival in an oxidative stress environment through antioxidation and c-met pathway activation. Sci China Life Sci.

[143] Deveza L, Choi J, Imanbayev G, Yang F (2012). Paracrine release from nonviral engineered adipose-derived stem cells promotes endothelial cell survival and migration in vitro. Stem Cells Dev.

[144] Nauta A, Seidel C, Deveza L, Montoro D, Grova M, Ko SH (2013). Adipose-derived stromal cells overexpressing vascular endothelial growth factor accelerate mouse excisional wound healing. Mol Ther.

[145] Cho HM, Kim PH, Chang HK, Ym S, Bonsra K, Kang BJ (2017). Targeted genome engineering to control VEGF expression in human umbilical cord blood-derived mesenchymal stem cells: potential implications for the treatment of myocardial infarction. Stem Cells Transl Med.

[146] Dey ND, Bombard MC, Roland BP, Davidson S, Lu M, Rossignol J (2010). Genetically engineered mesenchymal stem cells reduce behavioral deficits in the YAC 128 mouse model of Huntington's disease. Behav Brain Res.

[147] Mei SH, McCarter SD, Deng Y, Parker CH, Liles WC, Stewart DJ (2007). Prevention of LPS-induced acute lung injury in mice by mesenchymal stem cells overexpressing angiopoietin 1. PLoS Med.

[148] Petrakis S, Raskó T, Mátés L, Ivics Z, Izsvák Z, Kouzi-Koliakou K (2012). Gateway-compatible transposon vector to genetically modify human embryonic kidney and adipose-derived stromal cells. Biotechnol J.

[149] Rudick RA, Ransohoff R, Lee J, Peppler R, Yu M, Mathisen P (1998). In vivo effects of interferon beta-1a on immunosuppressive cytokines in multiple sclerosis. Neurology.

[150] Xie C, Yang Z, Suo Y, Chen Q, Wei D, Weng X (2017). Systemically infused mesenchymal stem cells show different homing profiles in healthy and tumor mouse models. Stem Cells Transl Med.

[151] Jiang X, Fitch S, Wang C, Wilson C, Li J, Grant GA (2016). Nanoparticle engineered TRAIL-overexpressing adipose-derived stem cells target and eradicate glioblastoma via intracranial delivery. PNAS.

[152] Schug C, Urnauer S, Jäckel C, Schmohl KA, Tutter M, Steiger K, et al. TGFB1-driven mesenchymal stem cell-mediated NIS gene transfer. Endocr Relat Cancer. 2019;26:89–101. 10.1530/ERC-18-0173 30121623

[153] Wen D, Peng Y, Liu D, Weizmann Y, Mahato RI (2016). Mesenchymal stem cell and derived exosome as small RNA carrier and Immunomodulator to improve islet transplantation. J Control Release.

[154] Kojima R, Bojar D, Rizzi G, Charpin-El Hamri G, El-Baba MD, Saxena P (2018). Designer exosomes produced by implanted cells intracerebrally deliver therapeutic cargo for Parkinson’s disease treatment. Nat Commun.

